# Living Alongside an Eating Disorder: A Qualitative Exploration of the Experiences of People in the UK Who Have an Adult Family Member Living With an Eating Disorder

**DOI:** 10.1192/bjo.2024.148

**Published:** 2024-08-01

**Authors:** Laura Gill

**Affiliations:** University of Edinburgh Medical School, Edinburgh, United Kingdom

## Abstract

**Aims:**

Eating disorders do not only affect the person who is suffering, but also their family. This qualitative study aims to understand the impacts on individuals who have an adult family member with an eating disorder, and what support they seek for their own well-being.

**Methods:**

A convenience sample of 11 volunteer participants (10 family members and 1 charity worker) from the United Kingdom (UK) were recruited for interviews. Four UK eating disorder charities assisted with outreach by sharing the Participant Information Sheet to their service users. Semi-structured interviews were conducted between February and March 2023. Interviews lasted on average 54 minutes and were recorded on video call (n = 9) or face-to-face on the University of Edinburgh campus (n = 2). Transcripts were analysed using thematic analysis, following a grounded-theory constructivist approach.

**Results:**

Participants discussed how their lives were changed by engaging with the care of their family member, leading to a shift in family dynamics and a change in understanding of what it means to be a ‘normal’ family. Most interviewees perceived their relative's eating disorder as a negative disruption to their own life, however one participant said that it had no negative impact on his well-being at all. Conflict in the household was a regular outcome, with four interviewees all using the phrase “treading on eggshells”. Siblings of adults with an eating disorder were described by their parents as being excluded from the family due to the ongoing parental focus on the healing of their child. Some participants accessed support groups and social media to connect with other families working through similar challenges.

**Conclusion:**

Having an adult family member with an eating disorder impacts the wider social network of the family. This dissertation argues that the socially constructed meanings of ‘care’ and ‘normality’, alongside the social relations with people placed in similar positions, inform the lived experiences of these individuals. This study's newfound illness narrative of ‘normality’ theorises that some people do not strive to help their relative with an eating disorder because it has already constructed the meaning of their normal life. Future research should aim to recruit a greater variation of participants, including more fathers, children, and siblings. This work endeavours to increase awareness of the support which families need during this time. It therefore opens the opportunity to consider how existing resources and services, both in healthcare and the third-sector, can be improved in the future.

